# Abstract of the Proceedings of the Buffalo Medical Association

**Published:** 1873-11

**Authors:** E. R. Barnes

**Affiliations:** Sec’y pro tem.


BUFFALO

### Medical and Surgical Journal.

VOL XIII. NOVEMBER, 1873. No. 4.

### Original Communications.

---:o:---

ART. I.—*Abstract of the Proceedings of Buffalo Medical Association.*

August 19, 1873.

At an adjourned meeting of the Buffalo Medical Association held Tuesday evening August 19, there were present: The President Dr. Hauenstein in the chair, and Drs. White, Gay, Cronyn, Sloan, Brecht, Wetmore, Samo, Briggs, Strong and Barnes.

The Secretary being absent, Dr. Barnes was elected Secretary pro tem.

Dr. Hauenstein stated that the subject for discussion was Food for Infants. This subject was little understood by the community. Thousands of infants were the victims of improper food. Cholera Infantum was a frequent result, and if Physicians had the power of informing the public as to the quality and quantity of food proper for infants much good would result.

Dr. White said that his views had long been settled on this subject which was one of much importance. The best food was that provided by Nature in the form of the Mother's Milk—but there were various reasons why this was not always available, as the death of the mother, agalactia, the occurrence of one of the forms of fever, of menstruation and of pregnancy. Then it becomes necessary to find a substitute, and the best is furnished by a Wet Nurse. But wet nurses are not made to order and often one cannot be obtained; or if one is found she must deprive her own child of its natural food, so that still we must look for a substitute: Asses milk most closely resembles that of the human female and consequently ranks first as a substitute, but this is difficult to obtain, and the same objection applies, though perhaps in a less degree to goat's milk, which is next in merit. The most available milk is that of the cow, and it is better than any of the preparations which are manufactured, or gruels, broths, arrow root, or even than condensed milk. What are the ingredients of the Mother's Milk. It has a specific gravity of 1032. West, Becquerel and others give as the analysis of the solids in 1000 parts of this milk.

| | |
|---|---|
| Sugar | 43.64 |
| Casein | 39.24 |
| Butter | 26.66 |
| Salts | 1 |

from which it appears that 110 to the 1000 or more than 10 per cent. are solid matters. According to the same authorities cows milk has a specific gravity of 1033, and in every 1000 parts there are of solid constituents:

| | |
|---|---|
| Sugar | 38 |
| Casein | 55 |
| Salts | 6 |
| Butter | 15 to 45 parts, or |

from more than 11 to more than 14 per cent. of solid matter. But these constituents vary much under diverse conditions. The milk of different breeds of cows differ much, e. g. the Alderneys, Jerseys and Ayershires yield a much larger proportion of butter than the common cow. Close confinements of the animals in stalls, with lack of pure air and exercise, and improper food exert a deleterious influence on the milk, by diminishing all its solid constituents, by increasing its tendency to become acid, and in other ways.

In Cities the people generally cannot be sure that the milk with which they are supplied is perfectly pure, and obtained from cows that have been properly fed and cared for, but there are certain simple means within the reach of all, which are of value in determining the quality of the milk: such as litmus paper, to ascertain its reaction, whether acid or alkaline, instruments for determining its specific gravity, which are sold cheaply; and test tubes, or vials, in which the milk may be allowed to stand until the quantity of cream which rises to the top shall inform us as to its richness in fatty matters.

It will be seen by the above analyses that in cow's milk, casein exists in larger proportion than in that of the human female, sugar in less, while the quantity of butter varies greatly according to the breed of the cow. Now, how can we best imitate the mother's milk, from the milk of the cow? In the first place the product of a single cow, with a young calf, or one not far from the age of the infant, is best; then, the cow should be milked frequently, two at least, or three times daily. The milk should be placed in a cool situation and allowed to stand an hour or so—after which the upper one half of the milk may be taken for use. This upper portion now contains a *diminished* quantity of casein, which has partly gravitated downwards, and the fatty matters have tended upwards, while the milk has been standing. Dilute with water, say one third of water to two thirds of milk, and add a little sugar; brown sugar if the infant is constipated, white, if its bowels are relaxed, or we may add sugar of milk.

The child should always be fed from a bottle, because it calls into play certain muscular actions, and stimulates to activity the salivary glands. It is nature's method. The bottle should be kept scropulously clean in every part. The best plan is to have two bottles, one of which may be cleansed while the other is in use. Never feed from one bottle, but once until it has been again cleaned, and the milk should be prepared only at the time when it is to be used. The temperature of the milk should be from 96° to 100° Fahrenheit, that is about the temperature of the milk of the mother.

Milk thus prepared contains all the elements of the mother's milk. It contains all the elements necessary for growth, and warmth or calorification. Its casein is closely assimilated to the albumen of the blood, in digestion, while its butter and sugar contribute to the formation of fat, and to the generation of heat by resolution into their elements of carbonic acid and water. This is not true of other forms of food, meats being deficient in some elements, and vegetables or cereals in others. And here it may be remarked, parenthetically, that the peculiar constitution of milk renders it the best article of diet for the adult sick, especially in the fevers, a fact which is often too lightly esteemed.

Never use acid milk, and do not add sugar to correct this state as it will only make it worse. An alkali may be added, if indicated as lime water, soda, or if there is constipation, carbonate of magnesia As to the daily quantity to be given there is no absolute rule; it may be two pints daily during the first month, and at three months, thirty-six or forty-eight ounces. But there is no danger of giving too much if cold water is given to the child when thirsty, as nature readily and easily rejects a superabundance. If the stomach is too full it simply spills over.

Vogel suggests than the curd of mother's milk is less solid than that of cows. Limewater diminishes the tendency of casein, to coagulate and boiling the milk produces the same effects.

This diet should be continued until the fifth, sixth, or seventh month, or until the appearance of the incisors indicates a certain progress in the development of the digestive organs. Then we may supplement the milk diet, with Liebigs food for infants, old dried bread crumbs, boiled flour, barley water, animal soups, sago and beef tea, beef tea and rice, and at 8 or 9 months the infant may suck beef steak. No form of alcohol, ordinarily, should be used. When the molars have appeared, mixed food may be given, though the diet should still consist largely of semi-fluid substances. This is the time when a healthy child which is nursing may be weaned.

Dr. White also spoke of the importance of ventilation and cleanliness, and referred to the great number shown by statistics to die, in cities and hospitals, of those who are artificially fed. He criticised some of the rules published by the New York Board of Health, and considered those eminating from the obstetrical society of Philadelphia to be much more physiological. He thought it absurd to regulate arbitrarily the time for giving food; feed when hungry and give water when thirsty; mother is the best judge. Pure cream and raw meat are useful under some circumstances. Did not advise wearing thick flannel in hot weather. He agreed with Dr. Corson, of Philadelphia, that it was not over feeding, but insufficient alimentation, that was so fatal to infants. In conclusion the Dr. stated that condensed milk is often better than the milk which can he generally obtained in cities.

Dr. Gay congratulated the Society on the goodly number present and on the able opening, and thought it was well to compare notes on so important a subject. He was glad to be able to agree with all that had been said. There is a difficulty which often meets us, i. e., the physiological differences or peculiarities of infants. As in surgery the application which is grateful to one is painful to another patient with a similar lesion, so the food which is generally best for infants may in individual cases disagree. Hence, difference of views as to diet. What do statistics teach as to wet nurses. In France 51 or 52 per cent. of those nourished by wet nurses die, while only 12 per cent. of those which nurse mothers die. There are many objections to wet nurses on the score of character, constitutional vice, and their liability to require lager beer, porter &c., to stimulate the lacteal incretion. As an exceptional case only would recommend a wet nurse. Many preparations are manufactured, the best being "Mincasea," and "Prolactea." Of the domestic animals the cow meets our wants best, on the whole, but she should be purchased, fed and cared for as a horse. Prefers the Alderny breed.

The Dr. added that he supposed wet nursing to be fashionable. Mothers refrain from nursing their infants in order to devote themselves more to society. They are too exquisite to nurse; they have an abundant secretion of milk, but decline the maternal duty. Woud denounce such practice; which he thinks criminal and the penalty is often the loss of offspring.

Dr. Strong was interested in the remarks which had been made. Had adopted for some time the treatment of cow's milk, recommended by Dr. White, and agreed with him as to its value. But all circumstances must be favorable, in order to get the best results. If people can keep and properly treat a cow, all is well, but nine tenths of the citizens cannot. The practical difficulty is to obtain pure milk. It may be faulty because the cows have been stabled during the summer, and not exercised or watered properly, or the cow giving it may be pregnant, conditions which we cannot control. Hence he thought that a condensed milk might be provided, by taking the upper part of milk and condensing it. Again water used to dilute milk should always be boiled to destroy germs.

Dr. Barlett agreed with Dr. Strong. Cow's milk is the best, but the difficulties are almost insurmountable, of obtaining it pure for common use. The milk-man may not obtain it pure from the country, or it undergoes fermentation and change in transportation. Had been accustomed in his practice to prefer condensed milk. Would suggest as a practical remedy, for the evils complained of, the establishment of an Infant's Dairy, under proper supervision.

Dr. Cronyn. No one can speak with so much authority as Dr. White, as it has been his special duty to teach on this subject, but in a practical point of view I have a little the advantage of him, in my family at least—which has been large. All of my children, for whom I could obtain a wet nurse lived. In the cases where I could not it was not so well. In general practice have followed this course: when I could not obtain a wet nurse, or milk which was undoubtedly pure—it is questionable whether condensed milk is a good substitute—have resorted to one of the forms of nourishment of which we have a long list, as broths &c., and children will often thrive if judgment is used in selecting food best adapted to their peculiarities.

In respect to use of flannel I consider it very important. It is more essential that flannel, light, white flannel, should be worn in summer than in winter. It is much used in the tropics. Unlike cotton, it does not increase the normal heat of the body. Cholera infantum occurs in 999 cases in a thousand, between 12 o'clock P. M., and 2 o'clock A. M. After four months of age, the child kicks off its clothing, the skin becomes cold, the perspiration is checked and exosmotic action of the capillary system of the mucous membrane of the bowels results, which influencing the normal functions of the sympathetic, the intestinal disorder is induced. With proper use of flannel, this will be prevented. Retention of animal heat in a child is more important than in an adult.

Dr. Strong. I am in the habit of taking the child from the mother's breast not only in pregnancy &c., but when the mother is simply anæmic, and the child does not thrive.

Dr. Gay. Would like to hear from the bachelors.

Dr. Samo. Have nothing to add. Do not approve of feeding infants before appearance of teeth even if ten months old.

Dr. White. All the objections which have been raised have not been against the theory presented but arise from the difficulty of obtaining pure milk. But milk is after all the most available article we have. Dr. Bartlett's idea of an Infants Dairy is very good and should be acted on. The Dairy should be under the direction of the Board of Health or some competent authority. It would be a public boon and would pay. No objection to light flannel properly used.

Dr. Gay. Best food for infants when *traveling* is condensed milk.

Dr. Bartlett. Have for some time thought of the good to be accomplished by a well regulated Infants' Dairy. A committee from this Society should be appointed to superintend it.

The milk used to make condensed milk is obtained as at a cheese factory, being carefully tested before it is accepted. Condensed milk is not subject to the putrefactive charges which occur in milk as most people get it. On motion the Society adjourned.

E. R. BARNES, Sec'y pro tem.

